# Evaluation of integrin α_v_β_3_-targeted imaging for predicting disease progression in patients with high-risk differentiated thyroid cancer (using ^99m^Tc-3PRGD_2_)

**DOI:** 10.1186/s40644-022-00511-0

**Published:** 2022-12-19

**Authors:** Yiqian Liang, Xi Jia, Yuanbo Wang, Yan Liu, Xiaobao Yao, Yanxia Bai, Peng Han, Si Chen, Aimin Yang, Rui Gao

**Affiliations:** 1 Department of Nuclear Medicine, The First Affiliated Hospital of Xi’an Jiaotong University, Xi’an, China; 2 Department of Otolaryngology-Head and Neck Surgery, The First Affiliated Hospital of Xi’an Jiaotong University, Xi’an, China; 3 Foshan Atomical Medical Equipment Ltd, Foshan, China

**Keywords:** Integrin α_v_β_3_, Differentiated thyroid cancer, ^99m^Tc-3PRGD_2_, SPECT/CT, Disease progression

## Abstract

**Background:**

High-risk differentiated thyroid cancer (DTC) needs effective early prediction tools to improving clinical management and prognosis. This cohort study aimed to investigate the prognostic impact of ^99m^Tc-PEG_4_-E[PEG_4_-c(RGDfK)]_2_ (^99m^Tc-3PRGD_2_) SPECT/CT in high-risk DTC patients after initial radioactive iodine (RAI) therapy.

**Methods:**

Thirty-three patients with high-risk DTC were prospectively recruited; all patients underwent total thyroidectomy and received ^99m^Tc-3PRGD_2_ SPECT/CT before RAI ablation. Follow-up was done with serological and imaging studies. The correlation between ^99m^Tc-3PRGD_2_ avidity and remission rate for initial RAI therapy was evaluated using logistic regression analysis. The prognostic value of ^99m^Tc-3PRGD_2_ SPECT/CT was evaluated by Kaplan-Meier curve and Cox regression analysis.

**Results:**

^99m^Tc-3PRGD_2_ avidity was significantly correlated with the efficacy of initial RAI ablation and an effective predictor for non-remission in high-risk DTC (OR = 9.36; 95% CI = 1.10–79.83; *P* = 0.041). ^99m^Tc-3PRGD_2_ avidity was associated with poor prognosis in patients with high-risk DTC and an independent prognostic factor for shorter progression-free survival (PFS) (HR = 9.47; 95% CI = 1.08–83.20; *P* = 0.043). Survival analysis, which was performed between DTC patients with concordant (^131^I positive/^99m^Tc-3PRGD_2_ positive) and discordant (^131^I negative/^99m^Tc-3PRGD_2_ positive) lesions, indicated that patients with concordant lesions had significantly better PFS than those with discordant lesions (*P* = 0.022). Moreover, compared with repeated RAI, additional surgery or targeted therapy with multikinase inhibitors could lead to a higher rate of remission in ^99m^Tc-3PRGD_2_-positive patients with progressive disease.

**Conclusions:**

^99m^Tc-3PRGD_2_ SPECT/CT is a useful modality in predicting progression of the disease after initial RAI and guiding further treatment in high-risk DTC patients.

## Introduction

The incidence of thyroid cancer continuously increased over the past three decades [1]. Differentiated thyroid cancer (DTC) accounts for more than 90% of all thyroid cancers, and prognosis in the majority of DTC patients is excellent. However, increasing cases, especially those with high-risk DTC, have been reported to develop local recurrence or metastatic disease after initial surgery and radioactive iodine (RAI) ablation [2]. Two-thirds of these patients will never be cured with RAI therapy and become RAI-refractory (RAIR), with a 3-year survival rate of less than 50% [3]. Early identification of the propensity for disease progression after initial therapy in high-risk DTC patients can assist physicians to develop prompt and individualized treatment plans.

The routine evaluation of DTC patients includes the measurement of serum thyroglobulin (Tg), ^131^I scintigraphy, ultrasound, and computed tomography (CT). Due to positive thyroglobulin antibody (TgAb) or undifferentiated lesions that do not secrete Tg, serum Tg may not be a reliable predictor in some patients. ^131^I scintigraphy also often fail to detect lesions with impaired ability to concentrate iodine. CT and ultrasound provide only anatomic data, which may lag behind functional changes. Hence, ^18^F-fluorodeoxyglucose (FDG) positron emission tomography/computed tomography (PET/CT) is gradually being used to localize lesions in patients with suspected RAIR-DTC. However, due to the common co-existence of iodine-sensitive and -refractory disease in high-risk DTC, the relatively low glucose metabolism in the lesions with heterogeneous cells are likely to be missed on FDG PET/CT [4]. Moreover, enhanced glucose uptake in inflammatory tissues, such as reactive lymph nodes, reduces the specificity of ^18^F-FDG PET/CT [5, 6]. A more effective imaging method is needed for early detection of advanced diseases in high-risk DTC patients.

Integrin α_v_β_3_, which is significantly upregulated on several tumor cells and activated endothelial cells, plays essential roles in neoangiogenesis and tumor progression as a member of the arginine-glycine-aspartate (RGD)-binding subfamily [7]. Unlike ^18^F-FDG PET/CT, which is a diagnosis-only modality, RGD imaging provides not only a specific method for visualizing tumor angiogenesis but also therapeutic implications for antiangiogenetic and anti-α_v_β_3_ drugs [8–10]. ^99m^Tc-PEG_4_-E[PEG_4_-c(RGDfK)]_2_ (^99m^Tc-3PRGD_2_), a novel RGD peptide tracer, is specifically designed to recognize integrin α_v_β_3_. ^99m^Tc-3PRGD_2_ has been used to trace primary or metastatic lesions in patients with various tumors, including lung, breast, esophageal and thyroid cancers [11–14]. Our previous studies have validated ^99m^Tc-3PRGD2 was a valuable probe for the detection of recurrent lesions with negative radioiodine whole-body scintigraphy (WBS) [15]. In addition, integrin α_v_β_3_ has been reported to interact with the vascular endothelial growth factor receptor-2 (VEGFR-2) and platelet-derived growth factor receptor (PDGFR) [16, 17]. The cross-talk between integrin α_v_β_3_ and VEGFR-2/PDGFR is crucial for endothelial cell activation and angiogenesis. In vivo studies demonstrated that ^99m^Tc-3PRGD_2_ imaging was a noninvasive tool to predict and evaluate the response to therapy with antiangiogenic agents in breast cancer [8].

In the present study, for the first time, we analyze the utility of ^99m^Tc-3PRGD_2_ single photon emission computed tomography/computed tomography (SPECT/CT) for the prognostication of therapeutic effect and disease progression in patients with high-risk DTC after initial RAI ablation.

## Materials and methods

### Patients

A total of 33 patients with high-risk DTC being managed in the Department of Nuclear Medicine at the First Affiliated Hospital of Xi’an Jiaotong University between May 2017 and December 2020 were enrolled in this study. The cases should meet the following inclusion criteria: (i) patients who underwent total thyroidectomy and were prepared for RAI, (ii) histologically confirmed DTC (papillary and follicular thyroid carcinoma), (iii) defined as high-risk disease according to the 2015 American Thyroid Association (ATA) guidelines [18], that is the patients with gross extrathyroidal extension (ETE), incomplete tumor resection, distant metastases, postoperative serum Tg suggestive of distant metastases, pathologic N1 disease with any metastatic lymph node ≥3 cm in largest dimension, or follicular thyroid carcinoma with extensive vascular invasion (more than 4 foci of vascular invasion). The demographic parameters, including surgical summary and histopathology reports, of all the study participants were recorded. Serum Tg, TgAb and TSH values were measured by radioimmunoassay method. This study was approved by the ethic committee of the First Affiliated Hospital of Xi’an Jiaotong University. Written informed consent was obtained from all patients prior to their enrolment. This study has been registered at http://www.chictr.org.cn/ (No. ChiCTR1900028095).

### ^99m^Tc-3PRGD_2_ SPECT/CT imaging

^99m^Tc-3PRGD_2_ SPECT/CT and planar imaging were performed at 1 month after surgery and 1 to 3 days before RAI treatment using a dual-head gamma-camera (Discovery NM/CT 670 pro; GE Healthcare, Milwaukee, USA) with low-energy high-resolution collimators and a 20% energy window centered on 140 keV. The SPECT and coregistered spiral CT were performed at 40–60 min post injection of 740–1110 MBq (20–30 mCi) of ^99m^Tc-3PRGD_2_. SPECT scan (matrix 128 × 128 pixels, zoom 1.0, 30 s/frame/6°) of the neck and chest was performed with the patients’ arms raised above their head, followed by CT scan (120 kV, 160 mAs) with the same range of SPECT. The speed of whole-body planar scan (matrix 256 × 1024 pixels) was set at 18 cm/min. The imaging of each patient was reconstructed and analyzed using Xeleris 3.0 workstation (GE Healthcare).

### Image analysis and interpretation

The ^99m^Tc-3PRGD_2_ images were independently reviewed by 2 experienced nuclear medicine physicians who were blinded to the source, history and pathologic conditions of the patients. A positive lesion was defined as the uptake of radiotracer above its background, excluding physiologic uptake [7, 15]. Disease foci were divided into the following regions: thyroid bed (remnant/recurrent), nodal disease (cervical or mediastinal) and lung lesions. The number of lesions at each site was recorded, except in the lungs. When more than 5 lesions were detected in lungs, 5 lesions with the highest uptake were selected from each case for further analysis [4]. The tumor-to-background (T/B) ratio of the positive lesions on SPECT was measured and calculated by the same person using a consistent standard. Briefly, regions of interest (ROI) were drawn around the lesions with reference to integrated CT. And on the same section, a background ROI was set in the surrounding normal soft tissue. The T/B ratio was calculated by dividing the mean count of tumor ROI by the mean count of background ROI. In addition, the tumor volume of interest (VOI) for each lesion was calculated, and maximum standardized uptake value (SUV_max_) was defined as the maximum concentration in the target lesion (maximum radioactivity/volume of VOI))/(injected radioactivity/body weight). The detailed calculation of SUV_max_ was based on a patented algorithm (Patent No.: US11189374B2) [19].

### Follow-up and treatment intervention

After initial RAI and post-therapeutic WBS, the patients were followed up for an average of 21 months. Levothyroxine was administrated to all the patients to suppress serum TSH levels. The ultrasound of neck and unstimulated thyroglobulin (T4-Tg) level were examined every 3 to 6 months during follow-up visits. Additional CT and/or magnetic resonance imaging (MRI) was performed every 3 to 6 months in patients who demonstrated distant metastasis. Additional therapies like secondary surgery, RAI, or targeted therapy were recorded.

Based on the radiologic findings and serum Tg levels, disease status was classified as complete cure, clinical improvement, stable disease, or progressive disease [20–22]. Complete cure was defined as undetectable TSH-stimulated Tg levels (or < 2.0 μg/L) and in the absence of TgAb with no evidence of structural disease on imaging. Clinical improvement was defined as at least 25% reduction in serum Tg levels with ≥30% decrease in the cumulative diameter of lesions. Progressive disease was defined as an increase of at least 25% in the serum Tg levels, with > 20% increase in the sum of lesion diameters or 5 mm increase in the sum of lesion diameters or appearance of new lesions. Stable disease was defined as < 25% increase or decrease in the serum Tg level with no obvious change in the cumulative diameter of lesions (neither sufficient shrinkage for clinical improvement nor sufficient increase for progressive disease).

### Statistical analysis

Patients who achieved complete cure and clinical improvement were categorized to the remission group, while those with stable and progressive disease were categorized to the non-remission group. Multivariate logistic regression model was used to investigate factors that associated with non-remission. Progression-free survival (PFS), defined as the time interval from the date of initial RAI to the date of disease progression, was the primary end point of this study. Survival data were estimated by Kaplan-Meier analysis with the log-rank test, and Cox regression analyses for PFS were used to identify the significant prognostic factors in high-risk DTC. For patients with multiple ^99m^Tc-3PRGD_2_-positive lesions, the median of T/B ratio and SUV_max_ values was calculated to perform survival analysis based on semi-quantitative SPECT/CT parameters. A *P* values of < 0.05 were considered statistically significant on the basis of 2-sided testing. All statistical analyses were performed using SPSS 22.0 and GraphPad Prism v5.0.

## Results

### Patient characteristics

Baseline clinical characteristics of included patients are summarized in Table 1. Mean age was 44.5 years, and 19 (57.6%) were women. PTC was present in 29 (87.9%) patients, and the remaining 4 patients (12.1%) had FTC. Of these patients, 29 (87.9%) patients had lymph node metastasis, and 8 (24.2%) patients had lung metastases. Total thyroidectomy alone was performed in 1 patient (3.0%), 5 patients (15.2%) underwent total thyroidectomy with central neck dissection, 23 patients (69.7%) underwent total thyroidectomy with central and lateral neck dissection, and 4 patients (12.1%) received hemithyroidectomy followed by completion thyroidectomy with or without lymph node dissection. The range of serum TSH-stimulated Tg before RAI was < 0.16 - > 500 ng/ml (median: 65.4 ng/ml). Post-operatively, all patients received RAI therapy, and the doses of radioiodine ranged from 4.44 to 6.66 GBq (median: 4.81 GBq).

**Table 1:** Demographic and clinical characteristics

Characteristic	Number of patients (%)
Gender	
Female	19 (57.6)
Male	14 (42.4)
Age at diagnosis (years)	44.5 ± 15.3 (range: 19-73)
Histology	
Papillary	29 (87.9)
Follicular	4 (12.1)
TNM classification (AJCC 8th)	
T status	
T1	8 (24.2)
T2	7 (21.2)
T3	4 (12.1)
T4	12 (36.4)
Tx	2 (6.1)
N status	
N0	3 (9.1)
N1	29 (87.9)
Nx	1 (3.0)
M status	
M0	25 (75.8)
M1	8 (24.2)
Initial surgery	
TT only	1 (3.0)
TT + CND	5 (15.2)
TT + CND + LND	23 (69.7)
Hemithyroidectomy followed by completion thyroidectomy	4 (12.1)
Baseline Tg with TSH sitimulation (ng/ml) (in patients with negative TgAb)	65.4, < 0.16 - > 500^a^
Dose of initial RAI treatment (GBq)	4.81, 4.44–6.66^a^

Abbreviations: *TNM* tumor-node-metastasis, *AJCC* American Joint Committee on Cancer, *TT* total thyroidectomy, *CND* central neck dissection, *LND* lateral neck dissection, *Tg* thyroglobulin, *TSH* thyroid stimulating hormone, *TgAb* thyroglobulin antibody, *RAI* radioactive iodine

^a^Presented as median, range

### ^99m^Tc-3PRGD_2_ avidity correlated with non-remission and PFS of patients with high-risk DTC

^99m^Tc-3PRGD_2_ SPECT/CT was positive for disease in 25/33 (75.8%) patients and negative in 8/33 (24.2%) patients. The post-therapeutic WBS showed ^131^I avidity lesions in 14/33 patients (42.4%). Of the 33 patients, 12 (36.4%) patients achieved remission. Of the remaining 21 (63.6%) patients who did not achieve remission, 6 had stable disease and 15 had progressive disease. Univariate logistic regression analysis indicated that ^99m^Tc-3PRGD_2_ avidity was the risk factors predicting non-remission (OR = 9.50; 95% CI = 1.50–60.11; *P* = 0.017; Table 2). After adjusting for age, sex, pathologic type, clinical stage, TSH-stimulated Tg, and ^131^I uptake status, multivariate logistic regression showed that ^99m^Tc-3PRGD_2_ avidity was the independent risk factor predicting non-remission in patients with high-risk DTC (OR = 9.36; 95% CI = 1.10–79.83; *P* = 0.041; Table 2).

**Table 2:** Multivariate logistic regression of relationships between remission and non-remission patients’ features

Variable	Remission (*n* = 12)	Non-remission (*n* = 21)	Crude OR (95% CI)	*P*	Adjusted OR (95% CI)	*P*
Age						
< 55	10	13	1		1	0.408
≥ 55	2	8	3.08 (0.53–17.80)	0.209	4.07 (0.15–113.53)	
Sex						
Female	7	12	1		1	0.696
Male	5	9	1.05 (0.25–4.42)	0.947	1.44 (0.23–8.89)	
Pathologic type						
Papillary	11	18	1		1	0.473
Follicular	1	3	1.83 (0.17–19.90)	0.618	0.26 (0.01–10.10)	
Stage						
I + II	11	16	1		1	0.685
III + IV	1	5	3.44 (0.35–33.61)	0.289	0.45 (0.01–20.84)	
TSH-stimulated Tg						
≤ 50	7	6	1		1	0.279
> 50	5	14	3.27 (0.73–14.55)	0.120	2.87 (0.43–19.31)	
^99m^Tc-3PRGD_2_ uptake by metastases						
Not avid	6	2	1		1	0.041^*^
Avid	6	19	9.50 (1.50–60.11)	0.017^*^	9.36 (1.10–79.83)	
^131^I uptake by metastases						
Not avid	8	11	1		1	0.833
Avid	4	10	1.82 (0.42–7.94)	0.427	0.80 (0.11–6.12)	

Abbreviations: *OR* odds ratio, *CI* confidence interval, *Tg* thyroglobulin, *TSH* thyroid stimulating hormone

^*^P < 0.05

**Fig. 1:**
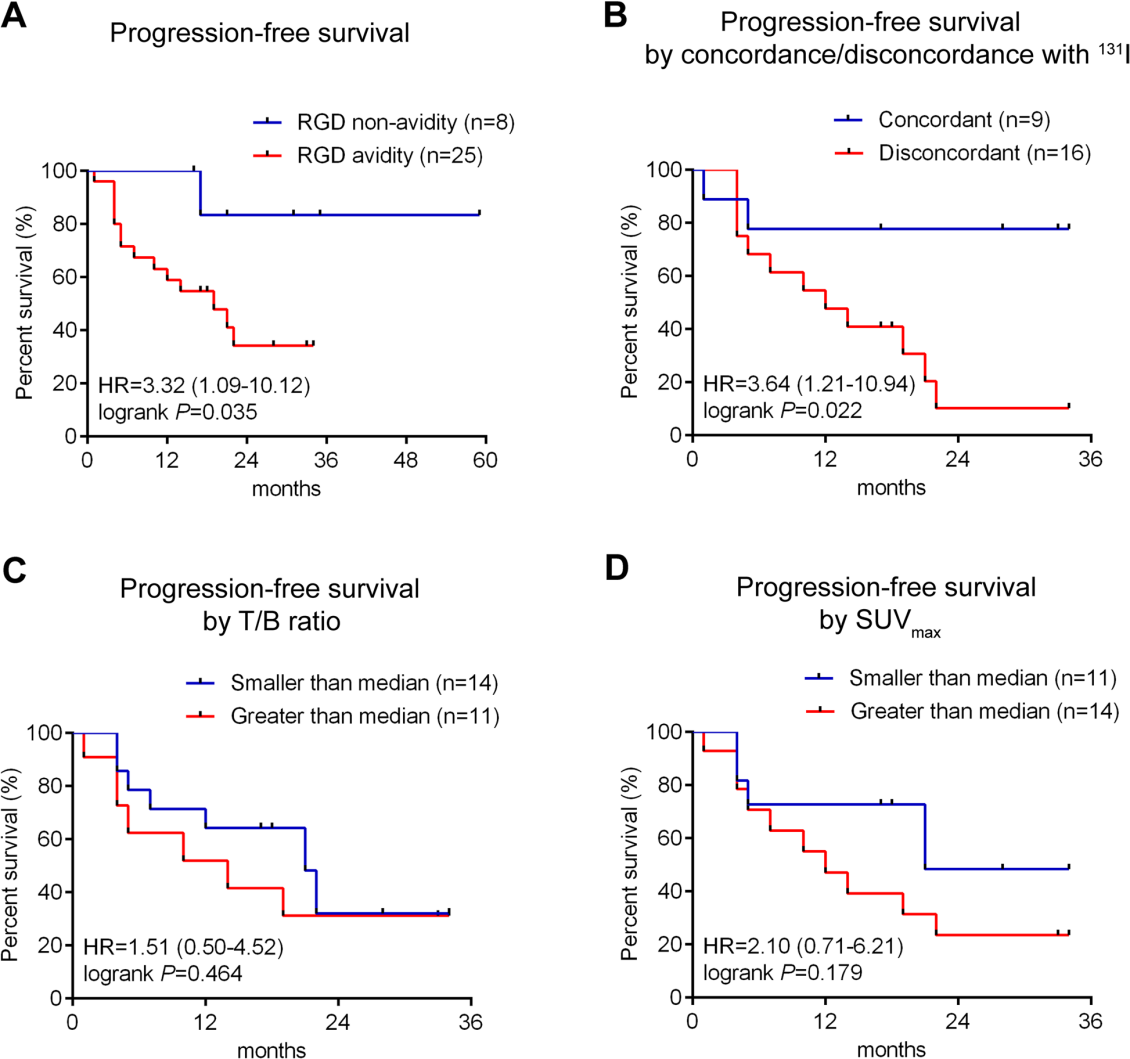
^99m^Tc-3PRGD_2_ avidity in high-risk DTC correlates with poor PFS of patients. **A** Kaplan-Meier plots of PFS in 33 patients with high-risk DTC stratified by the uptake status of ^99m^Tc-3PRGD_2_. **B** Kaplan-Meier plots for PFS in 25 ^99m^Tc-3PRGD_2_-positive patients stratified by concordance or discondance with ^131^I uptake. **C **and** D** Kaplan-Meier plots for PFS in 25 ^99m^Tc-3PRGD_2_-positive patients stratified by T/B ratio (**C**) or SUV_max_ (**D**). Abbreviations: HR, hazard ratio; T/B ratio: tumor-to-background ratio; SUV_max_: maximum standardized uptake value

**Fig. 2:**
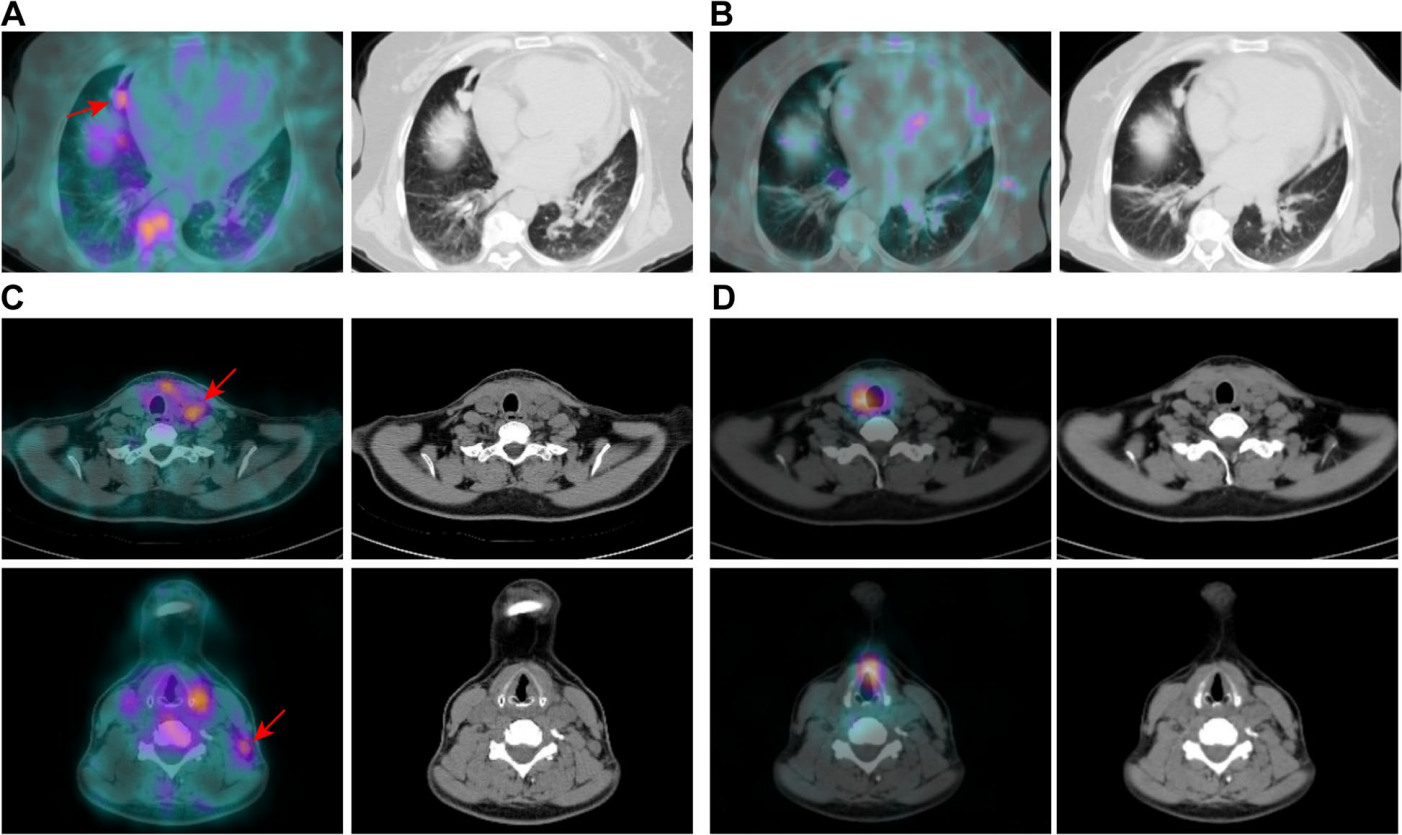
Representative ^99m^Tc-3PRGD_2_ SPECT/CT images of patients with ^131^I negative and ^99m^Tc-3PRGD_2_ positive lesions. **A** ^99m^Tc-3PRGD_2_ SPECT/CT detected lung metastases in a 63-year-old female PTC patient with initial TSH-stimulated Tg 3.1 ng/ml and positive TgAb. ^99m^Tc-3PRGD_2_ SPECT/CT showed a focal uptake in the right pulmonary nodule. **B** ^131^I post therapy WBS showed no uptake in this pulmonary nodule. **C** ^99m^Tc-3PRGD_2_ SPECT/CT detected lymph node metastases in a 41-year-old female PTC patient with initial TSH-stimulated Tg > 500 ng/ml. ^99m^Tc-3PRGD_2_ SPECT/CT showed focal uptakes in left cervical lymph nodes (level II and IV). The diffuse ^99m^Tc-3PRGD_2_ accumulation in thyroid bed was considered to be caused by postsurgical reactions. **D** ^131^I post therapy WBS showed no uptake in these lymph nodes. These neck lymph nodes were positive for metastatic PTC on histopathologic examination after secondary surgery

Kaplan-Meier survival curves revealed that ^99m^Tc-3PRGD_2_ uptake inversely correlated to PFS of high-risk DTC patients (*P* = 0.035; Fig. 1A). The median PFS of patients with ^99m^Tc-3PRGD_2_ non-avidity was not reached versus 19 months in patients with ^99m^Tc-3PRGD_2_ avidity. Multivariate Cox regression analysis showed that ^99m^Tc-3PRGD_2_ avidity was significantly associated with shorter PFS (HR = 9.47; 95% CI = 1.08–83.20; *P* = 0.043; Table 3). These results suggested that ^99m^Tc-3PRGD_2_ avidity was an independent risk factors for progression in high-risk DTC patients. Moreover, of the 25 patients with ^99m^Tc-3PRGD_2_-positive lesions, 9 patients showed ^131^I uptake in the lesions on the ^131^I post therapy scan, while 16 had no ^131^I uptake in the lesions. Figure 1B shows the Kaplan-Meier survival curve for PFS for concordant (^131^I positive/^99m^Tc-3PRGD_2_ positive) and discordant (^131^I negative/^99m^Tc-3PRGD_2_ positive) groups. The high-risk DTC patients with concordant lesions showed significantly better PFS than those with discordant lesions (*P* = 0.022; Fig. 1B). Median PFS was not reached in patients with concordant lesions while it was 12 months in the patients with discordant lesions. Representative ^131^I post-therapeutic WBS and ^99m^Tc-3PRGD_2_ SPECT/CT images of 1 of these patients with discordant lesions were showed in Fig. 2.

**Table 3:** Multivariate Cox regression analysis for progression-free survival in high-risk DTC patients

Variable	Number of patients	Progression (%)	HR (95% CI)	*P*
Age				
< 55	23	39.1	1	0.565
≥ 55	10	60.0	0.47 (0.04–6.17)	
Sex				
Female	19	52.6	1	0.776
Male	14	35.7	0.83 (0.22–3.10)	
Pathologic type				
Papillary	29	41.4	1	0.440
Follicular	4	75.0	2.33 (0.27–20.04)	
Stage				
I + II	27	40.7	1	0.839
III + IV	6	66.7	1.25 (0.15–10.79)	
TSH-stimulated Tg				
≤ 50	13	30.8	1	0.210
> 50	19	52.6	2.60 (0.58–11.57)	
^99m^Tc-3PRGD_2_ uptake by metastases				
Not avid	8	12.5	1	0.043^*^
Avid	25	56.0	9.47 (1.08–83.20)	
^131^I uptake by metastases				
Not avid	19	52.6	1	0.101
Avid	14	35.7	0.30 (0.07–1.26)	

Abbreviations: *HR* hazard ratio, *CI* confidence interval, *Tg* thyroglobulin, *TSH* thyroid stimulating hormone

^*^P < 0.05

### Relationship between disease progression with SPECT/CT parameters

^99m^Tc-3PRGD_2_-positive lesion sites and SPECT/CT parameters are represented in Table 4. ^99m^Tc-3PRGD_2_ SPECT/CT detected 2 thyroid bed lesions in 2 patients, and the median T/B ratio and SUV_max_ was 4.04 (range: 3.60–4.47) and 3.51(range: 3.26–3.76), respectively. Thirty-seven nodal lesions in 22 patients were detected, including cervical and mediastinal lymph nodes. The 37 nodal lesions had a median T/B ratio of 2.73 (range: 1.15–7.68) and a median SUV_max_ of 3.25 (range: 1.20–9.11). For lung lesions, five lesions with the highest T/B ratio from each patient were selected for the parameter analyses. The lung lesions in 5 patients had a median T/B ratio of 2.72 (range: 1.06–20.43) and a median SUV_max_ of 3.73 (range: 2.04–30.99).

**Table 4:** Sites of lesion detection and radiotracer uptake parameters on ^99m^Tc-3PRGD_2_ SPECT/CT

Site	Number of patients	Number of lesions	T/B ratio			SUV_max_		
			Median	Mean ± SD	Range	Median	Mean ± SD	Range
Thyroid bed	2	2	4.04	4.04 ± 0.61	3.60–4.47	3.51	3.51 ± 0.35	3.26–3.76
Nodal	22	37	2.73	3.05 ± 1.39	1.15–7.68	3.25	3.66 ± 1.85	1.20–9.11
Lungs	5	Multiple	2.72	4.50 ± 5.74	1.06–20.43	3.73	7.94 ± 11.34	2.04–30.99

Abbreviations: *T/B ratio* tumor-to-background ratio, *SUV*_max_ maximum standardized uptake value

The Kaplan-Meier method was used to estimate PFS probabilities based on whether T/B ratio or SUV_max_ was greater than, or less than median for each variable. There was a trend for improved PFS with T/B ratio or SUV_max_ less than the median (Fig. 1C and D). The median PFS of patients with T/B ratio greater than median was 14 months versus 21 months in patiens with T/B ratio less than median. In patients with SUV_max_ greater than median, the median PFS was 12 months versus 21 months in patients with SUV_max_ less than median. However, this trend did not reach the threshold of statistical significance, perhaps because of the small number of patients included.

### Managements of patients with ^99m^Tc-3PRGD_2_-positive lesions and progressive disease

**Fig. 3:**
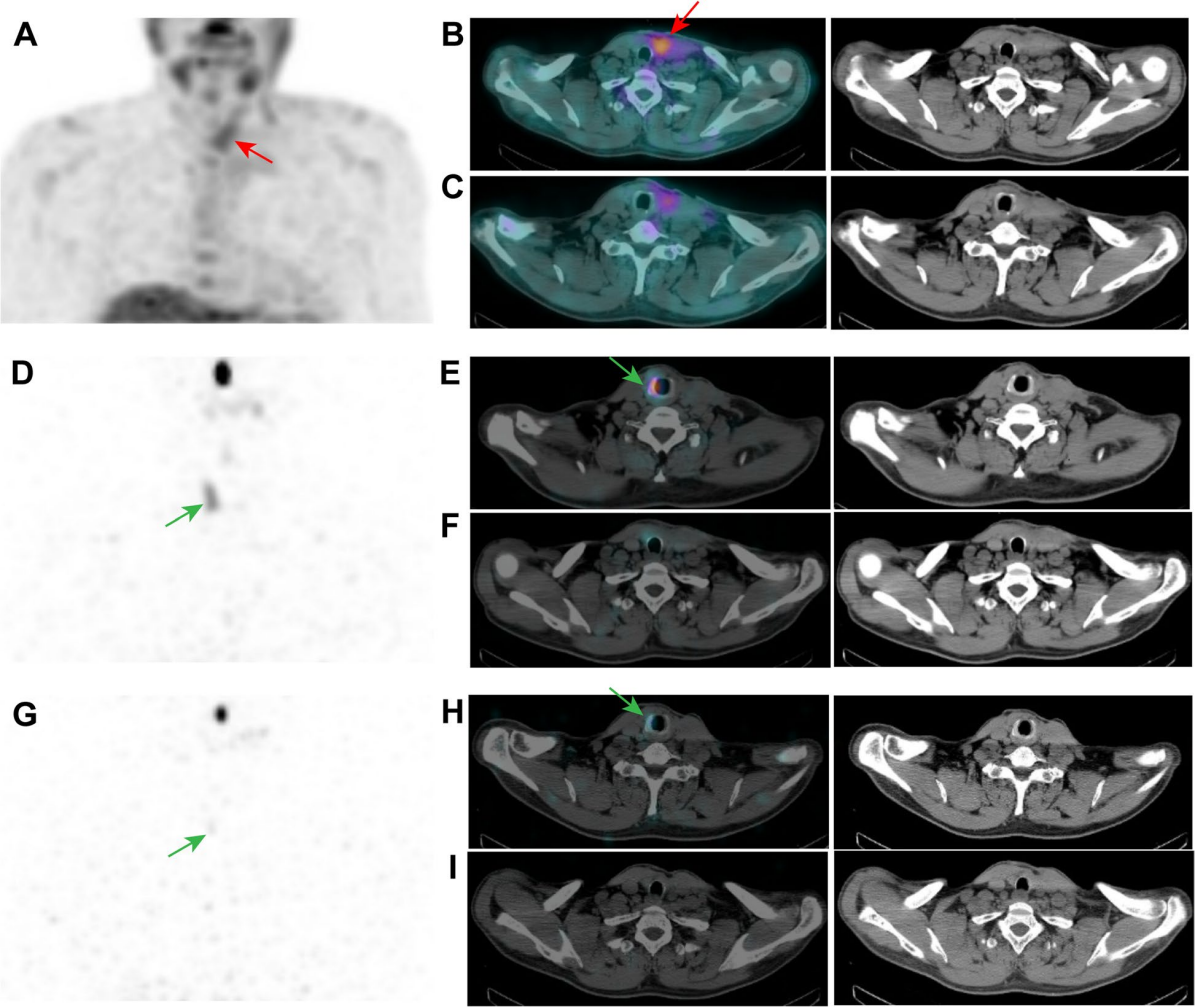
^99m^Tc-3PRGD_2_ SPECT/CT detected local recurrence in a 67-year-old male PTC patient with initial TSH-stimulated Tg 211.5 ng/ml. ^99m^Tc-3PRGD_2_ SPECT/CT before RAI showed a focal uptake in the left thyroid bed in planar image (**A**) and transaxial fused SPECT/CT (**B**), which was seen a soft-tissue mass on CT image. ^131^I post therapy WBS found a slight radioiodine uptake in planar image (**D**) and transaxial fused SPECT/CT (**E**) in the right thyroid bed. The lesion in the left thyroid bed was negative on ^131^I WBS (**F**). ^99m^Tc-3PRGD_2_ SPECT/CT showed no ^99m^Tc-3PRGD_2_ uptake in the right thyroid bed (**C**). Four months after initial RAI, the serum TSH-stimulated Tg of the patient elevated to 734 ng/ml. The neck ultrasound examination also detected local recurrence in the left thyroid bed. The patient received another RAI, and the post-therapeutic WBS showed only a vague uptake of radioiodine in the right thyroid bed (**G, H**). The lesion in the left thyroid bed was still negative on secondary ^131^I WBS (**I**)

After initial surgery and RAI ablation, 14 patients with ^99m^Tc-3PRGD_2_-positive lesions had progressive disease during follow-up. Of the 14 patients, 10 patients underwent additional surgery of the neck or lung because of lymph node or lung metastases, and malignant diseases were pathologically validated in resected lesions in 9 patients. As shown in Table 5, the result of the follow-up after additional surgery was remission in 6/10 patients, stable disease in 1/10 patients and progressive disease in 3/10 patients. Two cases received another RAI treatment. At the end of follow-up, progressive disease was observed in both patients who received second RAI. Representative ^131^I post-therapeutic WBS and ^99m^Tc-3PRGD_2_ SPECT/CT images of 1 of these 2 patients were showed in Fig. 3. Targeted therapy with multikinase inhibitors (MKIs) was applied in 2 patients. Of the 2 patients, one patient achieved clinical improvement, while another patient lost to follow-up.

**Table 5:** The management of patients with ^99m^Tc-3PRGD_2_ positive lesions and progressive disease after initial therapy and the results of the last follow-up

Disease status	Surgery (*n* = 10)	RAI (*n* = 2)	Targeted therapy (*n* = 2)
Complete cure	4	0	0
Clinical improvement	2	0	1
Stable disease	1	0	0
Progressive disease	3	2	0
Unknown	0	0	1

Abbreviations: *RAI* radioactive iodine

## Discussion

Thyroid cancer is clinically heterogeneous, varying from indolent to aggressively proliferative disease. Patients with high-risk DTC, which often represents less well-differentiated disease, have a lower chance of response to RAI therapy than low-risk patients [23]. More sensitive imaging modalities for identification of aggressive status is critical to the prognosis of high-risk DTC patients. RGD-peptide based SPECT/CT is a neo-angiogenesis imaging modality which has high affinity and specificity towards integrin α_v_β_3_. Xu et al. reported that ^99m^Tc-Galacto-RGD_2_ SPECT/CT had higher sensitivity than ^131^I WBS and morphological imaging in the detection of lymphatic and bone metastasis in DTC patients [7]. The overall sensitivity and specificity of ^99m^Tc-Galacto-RGD_2_ SPECT/CT were 92.86 and 86.36%, respectively, in the detection of metastatic DTC diseases. In our previous study, ^99m^Tc-3PRGD_2_ SPECT/CT showed high sensitivity in the detection of recurrence among DTC patients with Tg elevation but negative iodine scintigraphy (TENIS), and the sensitivity was improved to 100% in patients with TSH-stimulated Tg > 30 ng/mL [15]. However, there are few studies on the ability of RGD-based imaging to predict the prognosis after initial surgery and RAI in DTC patients. In this study, we found that ^99m^Tc-3PRGD_2_ avidity was an effective predictor for non-remission in high-risk DTC. The present study also found that ^99m^Tc-3PRGD_2_ avidity was associated with poor prognosis in patients with high-risk DTC. ^99m^Tc-3PRGD_2_ avidity was significantly correlated with PFS in multivariate analysis, which indicated ^99m^Tc-3PRGD_2_ avidity as an independent risk indicator for PFS in high-risk DTC.

In this study, about three-fourth of all patients (75.8%) had ^99m^Tc-3PRGD_2_-positive lesions at initial diagnosis before RAI treatment. Of the 25 patients with ^99m^Tc-3PRGD_2_-positive lesions, about two-third of patients (64.0%) had no ^131^I uptake in the lesions on ^131^I post therapy WBS, which would have been missed by standard RAI alone. Matching iodine- and ^99m^Tc-3PRGD_2_-positive lesions were observed in 9 patients. Survival analysis indicated that the presence of ^99m^Tc-3PRGD_2_ uptake in tumor lesions and the absence of ^131^I uptake in these lesions were significantly related to a worse PFS after initial RAI ablation. The role of ^99m^Tc-3PRGD_2_ SPECT/CT in therapy management of high-risk DTC was further observed in 14 ^99m^Tc-3PRGD_2_-positive patients having progressive disease after initial surgery and RAI. We found that additional surgery or MKIs therapy might lead to a higher rate of remission than repeated RAI in patients with ^99m^Tc-3PRGD_2_-positive lesions. Our results strongly suggested a linking between ^99m^Tc-3PRGD_2_ avidity and radioiodine refractory disease. This finding is consistent with prior studies by Zhao et al., which showed that RAIR metastatic lesions can be traced using ^99m^Tc-3PRGD_2_ SPECT imaging [14].

Nowadays, ^18^F-FDG PET/CT is the main method recommended by the ATA guidelines for the detection of RAIR-DTC. Many studies have demonstrated its utility in the detection of structural disease in RAIR-DTC patients with increasing sensitivity at higher levels of serum Tg, and changes of intermediate or high-risk patient management [24–26]. However, there are still some RAIR-DTC patients who have a negative ^18^F-FDG PET/CT, which drives the need for alternative imaging modalities. The PET or SPECT imaging of integrin α_v_β_3_ has recently been evaluated in refractory DTC, and some studies found that RGD-based imaging has better diagnostic performance than ^18^F-FDG [4, 27]. For instance, Parihar et al. reported ^68^Ga-DOTA-RGD_2_ PET/CT had higher specificity and overall accuracy than ^18^F-FDG PET/CT in detection of lesions in RAIR-DTC patients [4]. They noted that ^68^Ga-DOTA-RGD_2_ PET/CT and ^18^F-FDG PET/CT showed a similar sensitivity of 82.3%, however ^68^Ga-DOTA-RGD_2_ PET/CT had a higher specificity of 100% compared to 50% on ^18^F-FDG PET/CT. Our study indicated that compared with repeated RAI, additional surgery or targeted therapy with MKIs could lead to a higher rate of complete or partial remission in ^99m^Tc-3PRGD_2_-positive patients, suggesting ^99m^Tc-3PRGD_2_ scan could be able to guide the adjustments in management after the initial surgery and RAI ablation. Considering that the association of ^99m^Tc-3PRGD_2_ avidity with unfavorable prognosis, patients with a positive ^99m^Tc-3PRGD_2_ scan should be asked for a closer follow-up to detect recurrent or metastatic diseases in a timely manner. Therefore, ^99m^Tc-3PRGD_2_ SPECT/CT is a valuable diagnostic method for high-risk DTC patients and an effectively complementary modality for ^18^F-FDG PET/CT in refractory DTC.

In our study, further survival analysis revealed a trend towards worse PFS in patients with higher than median values for T/B ratio and SUV_max_. Recent studies reported the T/B ratios of ^99m^Tc-3PRGD_2_ in metastatic DTC lesions were positively correlated with growth rates of these lesions [14] and patients’ clinical stages [7]. Another study demonstrated that the SUV_max_ of RAIR-DTC lesions on ^68^Ga-DOTA-RGD_2_ PET/CT had a strong positive correlation with serum TSH-stimulated Tg levels, which reflecting the disease burden [4]. Hence, the parameters of RGD uptake could be used as potential imaging biomarkers for tumor burden and biologic aggressiveness. We did not detect a lineal correlation between Tg levels and ^99m^Tc-3PRGD_2_ uptake in this study. The possible reason is that the serum Tg was tested before initial RAI treatment, so that Tg was partly secreted by residual thyroid tissue, which could not reflect the real burden of recurrent or metastatic diseases.

In this study, the DTC patients with positive ^99m^Tc-3PRGD_2_ lesions all received full TSH suppression of less than 0.1 mIU/l with levothyroxine, but still had a high rate of disease progression. Thyroid hormone has been reported to increase tumor growth in various types of cancer, including hepatocellular, colorectal, and lung cancers [28–30]. A retrospective study followed 867 patients with intermediate- and high-risk DTC for a median of 7 years, documenting that patients with suppressed TSH levels were associated with worse 3-year overall survival [31]. The widespread use of TSH-suppressive therapy has recently been questioned, and individualized treatment based on each patient’s characteristics has been proposed. More recently, it was reported that integrin α_v_β_3_ has a high affinity binding site for thyroxine [32]. Thyroxine has been suggested to promote proliferation and angiogenesis in multiple cancer types via binding with integrin α_v_β_3_ [33, 34]. Therefore, TSH suppressive doses of levothyroxine in DTC patients with tumoral integrin α_v_β_3_ expression may have to be reconsidered, and integrin α_v_β_3_-targeting ^99m^Tc-3PRGD_2_ SPECT/CT could theoretically be utilized to identify these patients. Further mechanistic and clinical studies are needed to test this hypothesis.

There are still some limitations in this study. First, the follow-up was relatively short. Further studies with larger cohort of patients and longer follow-up period are required to validate our findings. Second, only 1 patient in this study underwent ^18^F-FDG PET/CT at 4 months after RAI ablation. A right cervical lymph node metastasis was visualized on both ^18^F-FDG PET/CT and ^99m^Tc-3PRGD_2_ SPECT/CT images, which was pathologically validated by fine needle aspiration biopsy. Future prospective research including parallel ^18^F-FDG PET/CT and ^99m^Tc-3PRGD_2_ SPECT/CT examinations is needed to clarify the effect of FDG PET/CT results on prognostic significance of ^99m^Tc-3PRGD_2_ SPECT/CT and compare the diagnostic and prognostic values of the 2 imaging modalities. Third, further experiments are indispensable to determine the underlying mechanism of integrin α_v_β_3_ promoting DTC.

## Conclusion

In this study, ^99m^Tc-3PRGD_2_ SPECT/CT was found to be a promising predictor for poor therapeutic effect and unfavorable prognosis after initial RAI treatment in patients with high-risk DTC. This imaging modality also contributed to the selection of therapy strategies and could been established as a standard procedure in the treatment of high-risk DTC patients.

## Data Availability

The data used to support the findings of this study are available from the corresponding author upon request.
